# Supplementing the Diet of Dairy Goats with Dried Orange Pulp throughout Lactation: I. Effect on Milk Performance, Nutrient Utilisation, Blood Parameters and Production Economics

**DOI:** 10.3390/ani11092601

**Published:** 2021-09-04

**Authors:** José Luis Guzmán, Ignacio Martín-García, Alejandro Pérez-Écija, Manuel David García-Brenes, Luis Ángel Zarazaga, Manuel Delgado-Pertíñez

**Affiliations:** 1 Departamento de Ciencias Agroforestales, Escuela Técnica Superior de Ingeniería, Universidad de Huelva, “Campus de Excelencia Internacional Agroalimentario, ceiA3”, Campus Universitario de la Rábida, Carretera Huelva-Palos de la Frontera, s/n., 21819 Palos de la Frontera (Huelva), Spain; guzman@uhu.es (J.L.G.); zarazaga@uhu.es (L.Á.Z.); 2 Estación Experimental del Zaidín (CSIC), Profesor Albareda 1, 18008 Granada, Spain; ignacio.martin@eez.csic.es; 3 Departamento de Medicina y Cirugía Animal, Campus Rabanales, Universidad de Córdoba, 14104 Córdoba, Spain; alejandro.perez.ecija@uco.es; 4 Departamento de Economía Aplicada II. Escuela Técnica Superior de Ingeniería Agronómica, Universidad de Sevilla, Carretera de Utrera Km 1, 41013 Sevilla, Spain; mdgarcia@us.es; 5 Departamento de Agronomía, Escuela Técnica Superior de Ingeniería Agronómica, Universidad de Sevilla, Ctra. Utrera Km 1, 41013 Sevilla, Spain

**Keywords:** Payoya breed, citrus byproduct, digestibility, rumen fermentation, complete lactation, blood chemistry, economic profit

## Abstract

**Simple Summary:**

Dried orange pulp can conveniently replace cereals in ruminant diets; however, no published reports have considered a similar substitution for the goat diet throughout lactation. Therefore, in this study, we evaluated the effects of cereal replacement (40% and 80%) by dried orange pulp in the diet of Payoya breed goats during the entire lactation period (180 days) on milk yield and composition, blood metabolites and production economics. Also evaluated, in mid-lactation, were the nutrient utilisation and ruminal fermentation of the dried orange pulp diets. Milk production and gross composition did not change in goats fed diets that included dried orange pulp. A decrease in nitrogen availability and retention was observed, while an increase detected in the digestibility of cellulose likely boosted rumen microbial protein synthesis. No pathological effects of dried orange pulp supplementation were detected in blood metabolites. The partial substitution of cereals for dried orange pulp reduced costs and raised economic profits. In conclusion, the partial replacement of cereals by dried orange pulp is a profitable and healthy nutritional strategy in dairy goats that does not compromise their productivity and is suitable for the entire lactation period.

**Abstract:**

Dried orange pulp (DOP) can be incorporated into ruminant diets, but no reports have considered this strategy during the entire lactation period in goats. Two experiments were performed using lactating Payoya goats. In experiment 1, to study the effect, over 180 days, of DOP on milk yield and composition, blood metabolites and economic values, 44 primiparous goats were allocated into three groups: control diet (concentrate plus lucerne) and DOP40 and DOP80 diets, in which DOP replaced 40% and 80%, respectively, of the cereals. Nutrient digestibility and rumen fermentation were also studied (experiment 2). The DOP diets did not affect milk yield and composition. DOP triggered lower intake and digestibility of ether extract and crude protein. Ruminal fermentation was unaffected by DOP, except for a decrease in butyrate for DOP80. The energy balance was unaltered by diet while the balance and retention of nitrogen decreased. Regarding plasma biochemistry, DOP supplementation caused changes that could indicate an improvement in hepatic function and reduced muscular damage and oxidative muscular stress. Moreover, DOP80 provided a profit increase of EUR 3.27/goat. In conclusion, the partial replacement of cereals by DOP is a profitable and healthy nutritional strategy in dairy goats and is suitable for the entire lactation period without compromising productivity.

## 1. Introduction

Enormous possibilities exist for using agroindustrial byproducts in animal feed if productivity or product quality is not compromised. The use of byproducts could eliminate environmental pollution problems and expenses derived from their management and disposal, while reducing livestock dependence on raw materials that can be consumed directly by humans, contributing to the sustainable use of biological resources. For example, Romero-Huelva et al. [1] concluded that the mixture of tomato fruits, citrus pulp, brewer’s grain and brewer’s yeast could replace 47% of conventional ingredients (corn, wheat bran, sunflower meal and soy flour) in the concentrate of the dairy goat diet, reducing feeding costs and methane production and leading to a healthier fatty acid profile in milk without compromising nutrient utilisation or milk yield.

Orange pulp is a byproduct of the orange juice industry that can be used fresh, pressed, dried and in pellets. Spain is the largest producer of oranges in the EU and the sixth largest producer worldwide [2]. In 2018, the country produced 3.9 million tons [3], 94.7% of which was produced in Valencia and Andalusia. As the production rate is 0.63 tons of pulp per ton of oranges [4], Spain produced 2.4 million tons of orange pulp in 2018. Moreover, Spain is also a major producer of goat livestock, and in 2018, it produced the second-highest number of goats of any European country (2.76 × 10^6^) [2].

The main use of orange pulp is in rations for high-production ruminants since its nutritional value is similar to that of cereal grains. Including the pulp in ruminant feed as a total or partial substitute for cereals could also decrease the cost of food.

There are very few published works on the effects of feeding orange pulp on the milk yield of dairy goats. López et al. [5] and Ibáñez et al. [6] have verified, in short experimental periods during mid-lactation, that replacing corn or barley grain with citrus pulp in the diet of Murciano-Granadina goats did not affect food intake, digestibility of most nutrients, milk production or gross composition. Very recently, our group examined how replacing cereal concentrates with dehydrated orange pulp (DOP) in the diet of goats affected the milk performance and health status, but only during early lactation [7]. Our group demonstrated that DOP supplementation did not cause any pathological blood chemistry alteration in goats. Moreover, supplemented diets correlated with lower blood markers of muscular stress and damage during late gestation.

As a result of this preliminary work, our starting hypothesis is that DOP can partially replace cereals in the diet of the dairy goat over long term (early, mid and late lactation) dietary supplementation and would not detrimentally affect goat milk performance, health status or nutrient utilisation, and could be a practical and cost-effective alternative to cereals. Therefore, this work studies the effects of substituting cereal by DOP in the diet of dairy goats during the entire lactation period on dam milk yield and composition, blood metabolites and economic values. In addition, a second experiment was conducted in mid-lactation to evaluate the nutrient utilisation and ruminal fermentation of goats consuming the experimental diets.

## 2. Materials and Methods

Two in vivo experiments were performed using lactating Payoya goats at the experimental farm of the University of Huelva (Huelva, Spain). Experiment 1 studied the effect of replacing cereal with DOP in the diet of dairy goats during the entire lactation period (180 days) on dam milk yield and composition, blood metabolites and economic values. In addition, a short-term in vivo trial (experiment 2) was conducted mid-lactation using the same experimental diets to evaluate nutrient utilisation and ruminal fermentation.

### 2.1. Experimental Diets, Goats and Experimental Procedure (Experiment 1)

Forty-four primiparous goats were assigned to three experimental groups, each with a different diet; the groups were balanced according to the goats’ live weight (LW) and body condition scores (BCS) [8]. These goats had been included in a previous experiment that assessed the effect of DOP in the diet of dairy goats on milk yield and composition and blood parameters of dams and growth performance and carcass quality of kids [7]. The animals were housed in a communal pen, and each experimental group was separated by metal barriers with free access to a yard. The three experimental diets were as follows: control (CD, *n* = 14), fed a commercial concentrate plus alfalfa hay as forage; DOP40 (*n* = 16) based on CD, but with 40% of the cereals in the concentrate replaced by DOP; DOP80 (*n* = 14), based on CD, with 80% of the cereals in the concentrate replaced by DOP. After parturition, the animals were offered the experimental diets adapted to early-mid- and late lactation. The formulation of the rations and the chemical composition of the isoenergetic and isoproteic diets are shown in Table 1. Formulations were designed using the Feed Ration Balancer (Format Solutions) software, version 2.0 (2017; Cargill, Inc., Wayzata, MN, USA). Hay and concentrate samples were prepared for analysis in each lactation stage (early-mid- and late) by mixing equal amounts of the subsamples collected through the lactation period and storing them at 4 °C.

Goats were allowed ad libitum access to water and were fed once daily in the mornings. Each group’s daily food intake was calculated by subtracting the orts (uneaten food) from the amount of food offered. On average, goats were fed 0.4 kg of alfalfa hay and 1.88 kg (early lactation, until 60 d, see Guzmán et al. [7]) or 1.81 kg (mid-lactation, until 120 d) or 1.57 kg (end lactation, until 180 d) of concentrate per animal daily. After weaning, the total average intake per goat in each diet group was determined in early (DM was 2.04, 2.00 and 1.98 kg/d; crude protein was 0.36, 0.33 and 0.36 kg/d; and gross energy was 9.28, 8.90 and 8.74 Mcal/d in the CD, DOP40 and DOP80 groups, respectively), mid- (DM was 1.92, 1.92 and 1.89 kg/d; crude protein was 0.34, 0.32 and 0.35 kg/d; and gross energy was 8.76, 8.53 and 8.35 Mcal/d in the CD, DOP40 and DOP80 groups, respectively) and late lactation (DM was 1.78, 1.76 and 1.75 kg/d; crude protein was 0.32, 0.29 and 0.32 kg/d; and gross energy was 8.14, 7.83 and 7.75 Mcal/d in the CD, DOP40 and DOP80 groups, respectively). After weaning and coinciding with the test-day milk yield recordings, LW and BCS were recorded for all animals (early lactation, see Guzmán et al. [7]; mid-lactation, at 90 ± 5 and 120 ± 5 days postpartum; end lactation, at 150 ± 5 and 180 ± 5 days postpartum).

After weaning the kids, the dams were milked once a day (at 09:00) in a 12-stall Casse System milking parlour; individual recorder jars were used to measure the volume of milk produced. A repeated measures design was used for recording milk yield from each animal; test-day yields were recorded in early (see Guzmán et al. [7]), mid- (at 90 ± 5 and 120 ± 5 days postpartum) and end of lactation (at 150 ± 5 and 180 ± 5 days postpartum). The total production at 180 days postpartum was calculated according to the A4 method of the International Committee for Animal Recording [9]. Representative samples (aliquots from each animal to determine milk traits) were taken from the volumetric flask of the parlour during machine milking carried out at 120 ± 5 (mid-lactation) and 180 ± 5 (end lactation) days postpartum. The aliquots were placed in 50 mL plastic bottles with preservative (Azidiol, Panreac Química, Barcelona, Spain). Samples were refrigerated and immediately sent to the laboratory for analysis.

During the last month of lactation (150 to 180 days), blood from each animal (*n* = 10 for each diet treatment) was collected aseptically from the left jugular vein in fasting animals in the morning and poured into tubes containing lithium heparin. After centrifugation at 2300× *g* for 30 min, the plasma was frozen at −20 °C until analysis.

### 2.2. Goats and Experimental Procedure (Experiment 2)

Three months after partum (mid-lactation), six animals from each experimental group (CD, DOP40 and DOP80) were selected, according to their LW, BCS and milk production, for inclusion in a nutrient digestibility trial. For four days, these goats were individually housed in metabolic cages in the same stable with the other goats (from which they were separated by a fence) to mirror the conditions of the other animals as closely as possible and to provide visual, auditory and olfactory contact, to achieve better animal welfare [10]. On the first and fourth days, animals were weighed and the BCS was estimated [8].

Animals were fed once a day (9:00 h), and water was offered ad libitum. Refusals were weighed daily to determine the individual intake. The quantity of diet supplied was calculated in order to allow approximately 15% refusal. Samples of the supplied diet (2%) and refusals (30%) for each goat were collected daily, weighed and stored at 4 °C. At the end of the four experimental days, the samples were pooled and homogenised for later laboratory analyses.

The faeces of each goat were collected once each day at 9:00 h, weighed, and an aliquot (20%) was kept in plastic bags at −20 °C for later analysis of apparent digestibility. Ammonia loss through volatilisation was prevented by adding 20 mL 10% HCl (*v*/*v*) daily to the urine collecting receptacles. The volume and weight of urine from each animal was recorded daily, and aliquots (10%) were stored at −20 °C for later analysis of the energy and total N content, to determine the energy and nitrogen balances, respectively, and to measure purine derivative (PD) and creatinine concentrations. Before the analysis, frozen faecal and urine samples of each goat were defrosted, pooled and homogenised.

The goats were milked once a day using a portable milking machine (Mayfra, 4211, Valdelafuente, León, Spain), the individual milk yield was recorded, and an aliquot (10%) was stored at 4 °C. At the end of the fourth experimental day, milk samples were pooled, homogenised and stored at −20 °C until analysis.

On the last digestibility trial day, approximately 50 mL of rumen content was collected from each animal before the morning feeding, using an oral probe attached to a vacuum pump and strained through a nylon membrane (400 µm; Fisher Scientific S.L., Madrid, Spain) as described in Ramos-Morales et al. [11]. Two aliquots (1 mL) were taken and stored at −20 °C for, respectively, volatile fatty acids (VFA) and ammonia N (N-NH3) analyses.

### 2.3. Chemical Analyses of Feed, Faeces, Urine, Rumen Content, Milk and Blood

Apparent nutrient digestibility was determined from corresponding intakes and losses in faeces. Nitrogen and energy balances were obtained, accounting for N and gross energy losses in urine, faeces, and milk and energy lost in methane. Before analysis, the feed (experiments 1 and 2) and refusal (experiment 2) samples were oven-dried, and the faecal samples (experiment 2) were freeze-dried, and then all the samples were ground using a Wiley mill with a 1 mm screen. AOAC [12] methods were used to determine dry matter (DM, method 934.01), ash (method 942.05), ether extract (method 920.39) and N (method 984.13) content in the samples. Total N was determined by the Kjeldahl procedure and converted to crude protein (CP) by multiplying by a factor of 6.25. The analyses of neutral detergent fibre (NDF) and acid detergent fibre (ADF) were conducted according to Van Soest et al. [13]. Amylase was not used in the analysis of NDF, and both NDF and ADF were expressed as exclusive of residual ash. Acid detergent lignin (ADL) was determined through the solubilisation of cellulose with 72% sulphuric acid. Calcium and phosphorus content was determined by inductively coupled plasma-optical emission spectrometry (ICP-OES Jobin Yvon Ultima 2, Longjumeau, France), according to the manufacturer’s instructions. The gross energy (GE) content was determined using an adiabatic calorimeter (model AC600, LECO Corporation, St. Joseph, MI, USA), according to the manufacturer’s instructions. Sugar and starch, the feed units for milk (UFL) and digestible protein in the small intestine (PDI) were calculated using Feed Ration Balancer software.

In experiment 2, the methane output was calculated as in Aguilera [14] as 10.32% of digestible energy. In the urine samples, DM (weight in 6 g), ash (weight in 6 g), CP (weight in 10 mL) and GE (weight in 4 mL) were analysed following the same methods listed above. The PD and creatinine in the urine and VFA and N-NH_3_ analyses in the rumen content were performed following the corresponding procedures described by Arco-Pérez et al. [15].

In experiment 1, dry matter, protein, fat and lactose contents of milk were estimated by near-infrared spectroscopy (NIR) as described by Delgado-Pertíñez et al. [16], while somatic cell count (SCC, cells/mL) was measured by flow cytometry, using a Fossomatic Electronic Cell Counter [17]. In experiment 2, milk protein N content was calculated as the difference between total and non-protein N in milk (NPMN). Milk total N content was determined using the Dumas method (AOAC method 990.03), while the NPMN was analysed in samples of milk filtrates after precipitation with 15% (*w*/*v*) trichloroacetic acid solution [15].

In experiment 1, every blood parameter was measured using specific kits according to Tschuor et al. [18] and as described previously by Guzmán et al. [7]. The following analytes were determined: total proteins, albumin, total triglycerides, cholesterol, glucose, urea nitrogen, creatinine, total bilirubin, alanine aminotransferase (ALT), aspartate aminotransferase (AST), alkaline phosphatase, creatine kinase (CK), amylase, phosphorus, total calcium, magnesium, sodium, potassium and chloride concentration.

### 2.4. Economic Analysis

Profit was calculated using the following expression: total income − (feed costs + all other costs). A comparative analysis between the three groups’ diets was performed after expressing profit in three different and complementary ways: EUR/goat, EUR/day and EUR/litre of milk. Total income in each of the three groups was calculated by multiplying total milk production over the entire lactation period by the average sale price of the milk. This price was obtained from goat’s milk sales data published weekly by the Andalusian Regional Government’s Ministry of Agriculture and Fishing (Spain) from January 2019 to September 2020 [19]. The total feed costs throughout lactation were calculated by multiplying the feed inputs provided to each experimental group by their current market prices. Feed input prices were supplied by the Cargill Animal Nutrition company, while orange pulp pellet prices were furnished by Cítricos del Andévalo, S.A (Huelva, Spain). Other activity cost data (labour, animal health and energy) were provided by Cabrandalucía Federation, an organisation for the main goat breeding associations in the Spanish region of Andalusia.

### 2.5. Data Treatment and Statistical Analysis

Goats’ weight, BCSs and daily milk yield recorded in each stage of lactation (mid, at 90 ± 5 and 120 ± 5 days postpartum; late, at 150 ± 5 and 180 ± 5 days postpartum) were analysed using the repeated measures procedure. The model included the fixed between-subjects factors, dietary treatment (CD, DOP40 or DOP80) and prolificacy (single or double birth); the fixed within-subjects factor, month of lactation (repeated measures); and the interactions between these factors. For simplification, the results for the factor, month of lactation, in both repeated measures analyses have not been presented in this paper. The data for total milk yield from birth to 180 days postpartum and composition parameters of milk samples taken in each stage of lactation (mid, at 120 ± 5 days postpartum; late, at 180 ± 5 days postpartum) were analysed using a factorial model that included dietary treatment and prolificacy and the interactions between these factors as fixed effects. Plasma biochemical parameters were analysed using a factorial model that included dietary treatment and prolificacy and the interactions between these factors as fixed effects. For experiment 2, intake, digestibility, N and energy balance and rumen fermentation parameters were analysed by ANOVA, using the GLM (including the diet offered to the animals as a fixed factor). The data analysed were the average data obtained from the four days of confinement in the metabolic cages.

In all the statistical analyses, the individual goats were considered experimental units and the significance level was set at *p* ≤ 0.05. However, if 0.05 < *p* ≤ 0.10, a tendency for differences was defined. Tukey’s honestly significant difference (HSD) test was used where appropriate for pairwise comparisons of means. 

Statistical analyses were performed using IBM SPSS Statistics for Windows (version 25.0; IBM Corp., Armonk, NY, USA).

## 3. Results

### 3.1. Body Weight and Milk Yield and Composition

**Figure 1:**
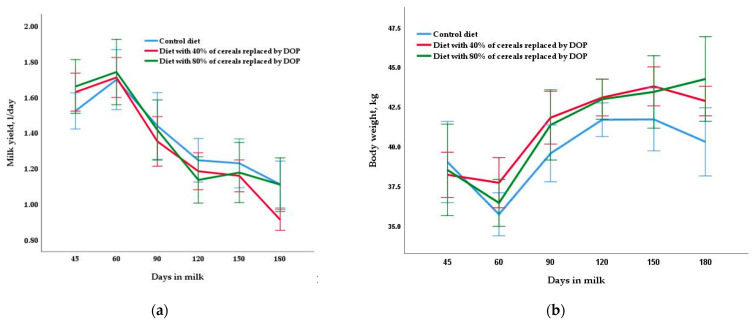
Changes throughout the lactation period (means ± SE) in milk yield (**a**) and body weight (**b**) due to dam diet treatments: control diet based on commercial concentrates plus alfalfa hay, diet based on concentrates with 40% of cereals replaced by dried orange pulp (DOP) plus alfalfa hay and diet based on concentrates with 80% of cereals replaced by DOP plus alfalfa hay.

Milk yield patterns and composition and the LW and BCS of goats during the entire lactation period, according to the treatment group, are presented in Table 2 and Figure 1. Live weight and BCS were similar for all three diets in mid- (40.9 kg and 2.76 on average, respectively) and late lactation (42.3 kg and 2.82 on average, respectively). Different diets did not change any milk yield variables examined (*p* > 0.05) (average daily milk yield in mid- and late lactation and total yield at 180 days were 1.29 L/day, 1.09 L/day and 253.2 L, respectively). Similar results were obtained for gross milk composition, although in late lactation, a trend (*p* < 0.10) was found towards a higher percentage of fat in the control and DOP40 diets than in the DOP80 diet, and a higher percentage of lactose in the DOP diets than in control diet. Milk yield was reduced at the end of the lactation (Figure 1), while the concentration of the variables related to the composition increased throughout the experiment.

### 3.2. Nutrient Utilisation and Ruminal Fermentation

The effects of the experimental diets on the nutrient intake and apparent digestibility during the metabolic cage trial are shown in Table 3. The intake of DM, OM, NDF and LAD did not differ among groups. The ether extract intake decreased significantly (*p* < 0.01) and the CP intake tended (*p* < 0.10) to decrease as the proportion of DOP increased. The ADF intake was higher (*p* < 0.05) for DOP80 than the control diet, while this diet showed higher and lower intake (*p* < 0.01), respectively, for hemicellulose and cellulose. The apparent digestibility of crude fat was lower (*p* < 0.05) as the DOP content increased in the diet, and CP digestibility was lower (*p* < 0.01) for the DOP diets. Lower (*p* < 0.01) digestibility of ADF and cellulose was found in animals fed the control diet, which, in contrast, showed higher (*p* < 0.01) digestibility of hemicellulose than DOP80.

The effects of experimental diets on the rumen fermentation parameters and purine derivates in urine are shown in Table 4. Apart from a tendency (*p* < 0.1) of the butyrate to be lower for DP80 than the control diet and purine derivates in the urine to be higher in the former diet, there were no differences related to creatinine in urine, total VFA concentration, the individual proportions of the different VFAs or ammonia N in the rumen.

Table 5 shows the impact of experimental diets on BW, energy and N balance as indicators of feed efficiency utilisation. Both BW and energy utilisation parameters (referred to BW) were not affected by the introduction of DOP. However, digestible N (*p* < 0.05) and N retention (*p* < 0.01) and, consequently, N balance (*p* < 0.01) were negatively affected by the introduction of DOP. Values for both the digestible N/intake N and the N balance/digestible N were significantly (*p* < 0.01) lower for DOP80. Only N balance/digestible N was lower for DOP40. However, milk N relative to both N digestibility and N intake was not affected by the treatment. The milk protein N/N balance rate was (*p* < 0.01) increased by DOP80. In addition, the digestible energy rate and the metabolisable energy as a function of energy intake were significantly (*p* < 0.05) lower for the DOP80 diet than for the DOP40 diet, but the latter was not different from the control diet for those parameters.

### 3.3. Clinical Blood Parameters

Biochemistry results are shown in Table 6. No significant differences between feeding groups were observed for total proteins, albumin, total triglycerides, cholesterol, urea nitrogen, creatinine, alanine aminotransferase, alkaline phosphatase, amylase, phosphorus, sodium, potassium or chloride. Creatine kinase (CK) and total bilirubin values were significantly lower (*p* < 0.05) in DOP40 and DOP80 groups compared to control (36% and 40% reduction for CK and 43% and 40% reduction for total bilirubin, respectively).

### 3.4. Economic Analysis

Table 7 presents the most important results of the economic analysis of the three experimental groups. Average incomes (EUR/goat) are highly similar. Compared to the control group, the DOP80 diet reduced average costs by EUR 1.86/goat, while the DOP40 diet yielded intermediate results. All of the other average costs were the same for the three experimental groups.

The average feed cost differences (EUR/goat) resulted in different profit margins for the three groups. Average EUR/goat profits expressed among the animals fed the DOP80 diet were EUR 41.27/goat while control group profits were EUR 38.00/goat, a difference of EUR 3.27/goat. Average EUR/goat/day profits were EUR 0.23 and EUR 0.21 for the DOP80 and control diets, respectively.

## 4. Discussion

### 4.1. Body Weight and Milk Yield and Composition

The use of DOP at the levels of inclusion tested does not appear to have detrimental effects on the milk yield or commercial milk traits. Therefore, the inclusion of DOP in well-balanced diets can replace cereals in the diet of dairy goats throughout lactation. As in our previous paper studying early lactation [7], the intake per animal obtained indirectly through the group’s consumption was similar in the different groups and phases of lactation. Moreover, the lack of influence of diet on LW and BCS and the comparable intake values obtained in the groups may be consistent with the lack of change observed in milk yield and composition. Similarly, in studies in which total cereal grain, corn [5] or barley [6] was replaced by DCP in mid-lactation Murciano-Granadina goats, intake, milk yield and gross composition were not affected by dietary treatments. The decrease in milk yield and the increase in macro composition values obtained in the three diets during the experiment are related to the normal progress of lactation, consistent with studies examining the same breed [20].

### 4.2. Nutrient Utilisation and Ruminal Fermentation

In their respective trials, Bueno et al. [21] and Oni et al. [22], when they substituted cereals in the diet of goat kids with citrus pulp at levels similar to those in our study, did not detect significant differences in DM consumption. However, Gholizadeh et al. [23] observed lower dietary intake in Iranian Saanen goats, even with very low levels of substitution of cereals by orange pulp (OP) (7−14%). Therefore, in our study with goats, and even more so because they are adult animals, the lack of (not uncommon) neophobia towards new foods can be considered highly positive since otherwise, significant losses in milk production could be induced.

In general, our results on nutrient utilisation are consistent with those summarised in the review of Bampidis & Robinson [24]. In those research studies, ruminant species fed dried citrus pulp were not impacted in terms of DM digestibility. Furthermore, they experienced a gradual decrease in fat digestibility and increases in the use of FAD as a response to increasing levels of citrus pulp. The authors argued that citrus pulp contains various energy substrates for rumen microflora, including soluble carbohydrates and readily digestible NDF that improve the utilisation of dietary fibrous fractions. Our results suggest that cellulolytic microorganisms could thrive in these conditions. Consistent with our finding of the linear effect of OP on ADFD shift are the results obtained by Bueno et al. [21], observing goat kids, and Martínez-Pascual and Fernández Carmona [25], studying lambs. Notably, regarding nutrient utilisation, several other studies observed an overall reduction of CP digestibility, supporting our calculations [24]. This observation could result from the low availability of the CP of OP due to high temperatures used in its drying or it could be that the ruminal environment promoted is not the most favourable for the proteolytic microorganisms.

The low ether extract and high ADF content of DOP resulted in significant differences among experimental diets for the digestibility of related nutrients (fat, hemicellulose and cellulose). Those differences, in general, were correlated with the substitution level (40 and 80%) of DOP and were in concordance with the results of Martínez-Pascual and Fernández Carmona [25] with wethers and lambs. To our knowledge, only Bhattacharya and Harb [26] obtained, in yearling lambs, a higher digestibility of crude fat as the proportion of citrus pulp in the ration was greater.

Our results on ruminal fermentation are similar to those compiled by Bampidis and Robinson [24]. The authors analysed nine studies in which different cereals were substituted by citrus pulp as energy sources with high pectin versus high starch content, and no decline in the total VFA concentration or shifts in the molar proportion individual VFA were observed. However, in contrast to our findings, most of those studies observed consistent decreases in the N-NH_3_ concentration. Regarding ruminal fermentation, the decrease in the molar proportion of butyrate may be the only deleterious effect of the incorporation of high proportions (80%) of DOP in the diet since this fatty acid constitutes one of the greatest energy resources for stimulation of epithelial cell proliferation in ruminants [27], which contributes directly to improving the efficiency of feed utilisation [28].

Barrios-Urdaneta et al. [29] examined the effects of supplementation with various proportions of barley grain or OP on digestion by sheep of ammonia-treated straw. They reported that urinary excretion of purine derivatives decreased linearly as the proportion of OP in the diet was increased, which is inconsistent with our observations. However, unlike in the present study, these authors did not measure the urinary concentration of creatinine as an indicator to validate the urine sampling and the analysis of the PD concentration, which would be critical since they observed differences among the experimental animals in the daily pattern of water intake and urine excretion. Thus, we could deduce that the excretion of microbial proteins in the rumen increased after detecting a 68% increase in the excretion of purine derivatives in the urine of animals fed DOP80. These results were validated with the values obtained for creatinine excretion, which are consistent with previous studies using goats [15,30,31], demonstrating that adequate total urine collection was achieved during trials and was performed in a standardised way. Finally, this observation could support the hypotheses of increased synthesis of cellulolytic microorganisms due to the inclusion of DOP. This inclusion would promote the observed increase in cell wall digestibility, and that could somehow compensate for lower CP digestibility, proven to maintain the productive parameters.

The control animals’ BW, energy and N balance values were within the range observed in previous works by our group [15,31,32] in dairy goats. Digestible N, N balance and N retained were negatively affected by the introduction of DOP into the diet. This reduction was especially pronounced for these last two parameters at the highest level of grain substitution. The type of diet had no impact on the balances of N in milk with respect to ingested, digested or retained N fractions, although a significantly lower protein to total N proportion was observed in the milk of the animals fed DOP80. Consistent with these results, Bhattacharya and Harb [26] in lambs and Martínez-Pascual and Fernández Carmona [25] in wethers and lambs also observed a decrease in N retention, especially at the highest inclusion levels (above 40%). However, to our knowledge, there are no comparable experiments with dairy goats, at least with inclusion levels as high as those tested in our trial, in which N retention is more relevant due to N secretion in milk. The lower CP digestibility of DOP diets compared to the control may explain the statistically significant lower rate of digested N relative to ingested N in animals fed those diets. In contrast, the balance of N with respect to digestible N declined gradually. The most marked decreases occurred as the proportion of DOP in the ration increased as a response to the shift observed in the CP digestibility combined with the relevant increase in urinary N in goats fed DOP80. Although with these results it would not be advisable, the substitution with DOP (especially at the level of 80%) to maintain the physiological status of the animal or the productive level, not having detected an evident impact on milk production throughout lactation, could suggest the positive long-term impact of DOP on the rumen fibrolytic microbes and fibre fermentative activity; however, further research should undoubtedly clarify DOP’s effect on rumen function.

In absolute terms, related to energy utilisation parameters, there were no effects of OP inclusion in the diet, digestible energy included. These results could be, to a certain extent, in disagreement with those of Bueno et al. [21], in which the digestibility of energy increased as the concentration of citrus pulp increased in the diet of kids, since, in our study, no changes occurred in the digestible energy rate/kg metabolic weight. Furthermore, no effects were observed for the ME/digestible energy and the proportions of milk energy with respect to digestible or metabolisable energy. However, the ratios of digestible energy or ME with respect to energy ingested were significantly higher for DOP40 than for DOP80, likely caused by a numerically lower energy intake in the animals that consumed the latter diet.

### 4.3. Clinical Blood Parameters

Every plasma biochemistry parameter was within reported reference values for healthy goats in every group [18,33]. Thus, no pathological effect was detected concerning these parameters due to dietary manipulation. As previously described [7], animals with both DOP diets showed significantly lower CK values compared to controls, which could be related to reduced muscular damage and oxidative muscular stress [34]. The significantly lower bilirubin concentrations in DOP groups compared to the control group could be related to improved hepatic clearance of this analyte and better bile duct function in these animals [35]. Contrary to results in early lactation [7], there were no differences between diets concerning glucose or total calcium concentrations in late lactation, which could be explained by differences in the homeostasis of these biomarkers between stages.

### 4.4. Economic Analysis

The results indicate no significant milk production differences among the goats fed the three different diets. Furthermore, replacing cereal concentrates with DOP in the goats’ feed reduced costs and raised profits slightly. The different average profit (EUR/goat) between the DOP80 group and the control group was quite small. This result is due, fundamentally, to the fact that at present, the price difference between DOP pellets and cereals is also small. Therefore, including DOP in goats’ diets has a limited economic effect, although it is extraordinarily important in the context of grain scarcity and high grain prices. Furthermore, a cereal substitute in the diet will always be of interest for other reasons, such as reducing environmental pollution and not competing with human food demands.

## 5. Conclusions

Our data suggest that DOP can be a valuable alternative to cereals in the concentrate fraction of diets for dairy goats during the entire lactation period without negatively affecting the milk yield and gross composition and the ruminal fermentation pattern or significantly impacting diet nutrient utilisation. The DOP also caused lower plasma levels of CK and bilirubin, implying reduced muscular oxidative damage and improved hepatobiliary functions. The study also demonstrated that DOP reduces costs and improves profit margins slightly in milk sales. Furthermore, as an alternative to standard goat feed, it is environmentally friendly.

## Figures and Tables

**Table 1:** Ration ingredients and proximate composition of the experimental diets ^1^ during the entire lactation period.

Items	Early and Mid-Lactation			Late Lactation		
	Control	DOP40	DOP80	Control	DOP40	DOP80
Ration ingredients, % dry matter (DM) basis						
Alfalfa hay	17.44	17.53	17.64	20.16	20.28	20.44
Concentrate						
Dehydrated orange pulp (pellets)	0	19.96	39.87	0.00	19.36	38.64
Grain oats	22.10	13.23	4.38	21.44	12.,83	4.24
Grain barley	8.53	5.11	1.70	8.28	4.96	1.65
Grain corn	19.34	11.61	3.89	18.76	11.25	3.77
Soy flour, 44%	7.31	10.23	12.97	7.09	9.92	12.57
Sunflower pellets, 28%	12.84	12.50	13.78	12.46	12.12	13.35
Grain peas	10.32	8.12	4.05	10.01	7.87	3.93
Salt	0.41	0.41	0.41	0.39	0.39	0.39
Stabilised lard	0.41	0.00	0.00	0.39	0.00	0.00
Vitamins and minerals ^2^	1.31	1.31	1.32	1.01	1.01	1.02
Proximate composition and nutritive value, % DM						
DM, %	87.17	87.61	88.05	87.08	87.08	88.09
Crude protein	17.76	16.59	18.43	20.92	18.66	18.30
Neutral detergent fibre	24.29	22.62	25.42	29.82	26.56	28.29
Acid detergent fibre	12.30	15.89	18.99	14.69	15.24	16.83
Acid detergent lignin	4.14	4.19	5.45	3.09	3.13	3.43
Sugar and starch	37.10	29.42	21.80	36.07	36.07	20.49
Ether extract	3.42	2.31	1.71	2.63	1.85	1.43
Ash	4.82	6.44	7.50	6.50	7.47	8.64
Calcium	0.42	0.80	1.02	0.60	0.96	1.27
Phosphorus	0.39	0.34	0.37	0.48	0.41	0.39
Gross energy, kcal/g DM	4.56	4.44	4.42	4.37	4.31	4.25
Feed units for milk, UFL/kg	0.98	0.98	0.97	0.98	0.98	0.96
Protein digestible in the intestine (PDI)	10.45	10.87	11.29	10.42	10.42	11.42

^1^ Control, diet based on commercial concentrates plus alfalfa hay; DOP40, diet based on concentrates with 40% of cereals replaced by dehydrated orange pulp (DOP) plus alfalfa hay; DOP80, diet based on concentrates with 80% of cereals replaced by DOP plus alfalfa hay; ^2^ Nutral cabras LD granulado, Cargill^®^, Barcelona, Spain.

**Table 2:** Milk performance during the entire lactation period of goats fed the experimental diets.

Item ^3^	Diet (D) ^1^			Prolificacy (PR)		SEM	*p* ^2^	
	Control	DOP40	DOP80	Single	Twins		D	PR
No. of goats	14	16	14	23	21			
BW in mid-lactation, kg	40.6	42.5	42.2	42.6	41.1	0.61	ns	ns
BCS in mid-lactation	2.73	2.80	2.77	2.81	2.72	0.03	ns	ns
BW in late lactation, kg	40.9	43.3	43.8	41.6	43.8	0.76	ns	†
BCS in late lactation	2.75	2.78	2.93	2.80	2.84	0.04	ns	ns
Milk yield (180 d), L	252.6	252.7	254.6	219.1	292.4	12.8	ns	**
Daily milk yield in mid-lactation, L/d	1.34	1.27	1.28	1.09	1.52	0.08	ns	**
Daily milk yield in late lactation, L/d	1.17	1.01	1.10	0.87	1.32	0.07	ns	**
DM in mid-lactation, %	12.2	12.3	12.4	12.4	12.2	0.11	ns	ns
DM in late lactation, %	13.8	14.0	13.1	13.8	13.5	0.20	ns	ns
Crude protein in mid-lactation, %	3.20	3.18	3.30	3.17	3.27	0.04	ns	ns
Crude protein in mid-lactation, g/d	41.8	37.8	40.7	33.3	47.4	2.33	ns	**
Crude protein in late lactation, %	4.32	4.05	3.98	4.03	4.17	0.11	ns	ns
Crude protein in late lactation, g/d	50.6	42.5	45.6	32.3	54.0	3.13	ns	***
Fat in mid-lactation, %	3.85	4.02	4.00	4.04	3.90	0.08	ns	ns
Fat in mid-lactation, g/d	51.3	48.0	48.6	42.4	56.6	2.73	ns	*
Fat in late lactation, %	4.83	4.72	4.02	4.61	4.43	0.16	†	ns
Fat in late lactation, g/d	55.7	47.2	45.5	37.8	57.6	3.86	ns	**
Lactose in mid-lactation, %	4.43	4.43	4.39	4.46	4.38	0.04	ns	ns
Lactose in late lactation, %	4.15	4.52	4.42	4.47	4.30	0.06	†	ns
LSCC in mid-lactation, cell/mL	5.64	5.28	5.54	5.47	5.48	0.08	ns	ns
LSCC in late lactation, cell/mL	5.78	5.35	5.58	5.62	5.45	0.09	ns	ns

^1^ control, diet based on commercial concentrates plus alfalfa hay; DOP40, diet based on concentrates with 40% of cereals replaced by dehydrated orange pulp (DOP) plus alfalfa hay; DOP80, diet based on concentrates with 80% of cereals replaced by DOP plus alfalfa hay; ^2^ statistical probability for comparisons: ns, not significant (*p* > 0.05); †, *p* ≤ 0.10; *, *p* ≤ 0.05; **, *p* < 0.01; ***, *p* < 0.001; no significant interactions between these factors were noted (*p* > 0.05).; ^3^ BW, body weight; BCS, body condition score; LSCC: Log_10_ somatic cell count.

**Table 3:** Effects of experimental diets ^1^ on nutrients intake and apparent digestibility.

Item	Diet ^1^			SEM	*p* ^2^
	Control *n* = 6	DOP40 *n* = 6	DOP80 *n* = 6		
Intake (g/day)					
Dry matter	1668	1591	1465	51.4	ns
Organic matter	1609	1514	1394	48.5	ns
Ether extract	50.9a	33.1b	22.2c	3.07	**
Crude protein	290	235	233	11.5	†
Neutral detergent fibre	467	398	407	13.4	ns
Acid detergent fibre	177b	219ab	253a	9.91	*
Acid detergent lignin	52.3	50.8	55.3	2.41	ns
Hemicellulose	290a	179b	154b	15.3	**
Cellulose	119b	168a	198a	9.07	**
Apparent digestibility (g/kg)					
Dry matter	763	784	749	3.65	ns
Organic matter	781	804	772	5.95	ns
Ether extract	908a	871b	763c	15.6	*
Crude protein	775a	715b	705b	10.6	**
Neutral detergent fibre	517	572	539	13.9	ns
Acid detergent fibre	327b	449a	456a	34.7	**
Hemicellulose	739a	722ab	671b	11.8	**
Cellulose	737b	846a	875a	17.3	**

Means with different letters (a, b, c) within each row differ significantly (*p* ≤ 0.05); ^1^ control, diet based on commercial concentrates plus alfalfa hay; DOP40, diet based on concentrates with 40% of cereals replaced by dehydrated orange pulp (DOP) plus alfalfa hay; DOP80, diet based on concentrates with 80% of cereals replaced by DOP plus alfalfa hay; ^2^ statistical probability for comparisons: ns, not significant (*p* > 0.05); †, *p* ≤ 0.10; *, *p* ≤ 0.05; **, *p* < 0.01.

**Table 4:** Effects of experimental diets ^1^ on the rumen fermentation parameters and purine derivates in urine.

Item ^3^	Diet ^1^			SEM	*p* ^2^
	Control *n* = 6	DOP40 *n* = 6	DOP80 *n* = 6		
Total VFA (mM)	73.8	80.9	78.1	8.3	ns
mol/100 mol					
Acetate	63.7	62.0	64.5	1.1	ns
Propionate	16.9	17.5	18.6	1.8	ns
Isobutyrate	0.508	0.224	0.732	0.116	ns
Butyrate	16.9ab	18.7a	14.1b	0.7	†
Isovalerate	1.36	0.985	1.62	0.22	ns
Valerate	0.502	0.610	0.553	0.082	ns
Acetate/propionate	4.24	4.33	4.20	0.54	ns
N-NH_3_, mg/L	288	280	299	22.1	ns
Purine derivates, μmol/kg BW^0.75^	573b	573b	963a	75	†
Creatinine, μmol/kg BW^0.75^	427	412	492	54	ns

Means with different letters (a, b, c) within each row differ significantly (*p* ≤ 0.05); ^1^ control, diet based on commercial concentrates plus alfalfa hay; DOP40, diet based on concentrates with 40% of cereals replaced by dehydrated orange pulp (DOP) plus alfalfa hay; DOP80, diet based on concentrates with 80% of cereals replaced by DOP plus alfalfa hay; ^2^ statistical probability for comparisons: ns, not significant (*p* > 0.05); †, *p* ≤ 0.10. ^3^ VFA, volatile fatty acids; BW, body weight.

**Table 5:** Metabolic weight (BW^0.75^), nitrogen and energy utilisation and feed efficiency in dairy goats fed the experimental diets.

Item	Diet ^1^			SEM	*p* ^2^
	Control *n* = 6	DOP40 *n* = 6	DOP80 *n* = 6		
BW^0.75^ (kg)	17.1	16.1	16.9	0.33	ns
BCS	2.83	2.79	2.79	0.07	ns
g/kg of BW^0.75^					
Digestible N ^3^	2.98a	1.67b	1.67b	0.072	*
N balance ^4^	0.990a	0.765b	0.609c	0.035	**
Retained N ^5^	0.507a	0.336b	0.102c	0.075	**
N utilisation (%)					
Digestible N/intake N	77.5a	71.5b	71.2b	1.03	**
N balance/digestible N	47.1a	42.3.1b	40.3c	2.14	**
Milk protein N/digestible N	21.0	22.9	27.6	1.40	ns
Milk protein N/N balance	45.3a	58.3a	70.6b	4.16	**
Milk protein N/milk total N	91.1b	91.0b	88.9a	0.320	*
Milk protein N/intake N	16.1	16.5	19.6	0.970	ns
Energy utilisation, MJ/kg of BW^0.75^					
Energy intake	2.21	2.19	1.93	0.076	ns
Faecal energy	0.523	0.479	0.488	0.021	ns
Urine energy	0.054	0.051	0.057	0.005	ns
Milk energy	0.174	0.191	0.185	0.005	ns
Digestible energy ^6^	1.69	1.71	1.44	0.061	ns
Methane energy ^7^	0.174	0.177	0.149	0.006	ns
ME ^8^	1.46	1.49	1.24	0.051	ns
Energy utilisation, %					
Digestible energy/energy intake	76.5ab	78.3a	74.4b	0.57	*
Milk energy/digestible energy	18.0	17.8	19.8	0.95	ns
Milk energy/ME	20.8	20.6	23.1	1.08	ns
ME/energy intake	66.2ab	67.8a	63.6b	0.61	*
ME/digestible energy	86.5	86.7	85.5	0.36	ns

Means with different letters (a, b, c) within each row differ significantly (*p* ≤ 0.05); ^1^ control, diet based on commercial concentrates plus alfalfa hay; DOP40, diet based on concentrates with 40% of cereals replaced by dehydrated orange pulp (DOP) plus alfalfa hay; DOP80, diet based on concentrates with 80% of cereals replaced by DOP plus alfalfa hay; ^2^ statistical probability for comparisons: ns, not significant (*p* > 0.05); *, *p* ≤ 0.05; **, *p* < 0.01; ^3^ digestible N = intake N—faecal N; ^4^ nitrogen balance = digestible N—urine N; ^5^ retained N = N balance—milk total N; ^6^ digestible energy = energy intake—faecal energy; ^7^ methane energy = methane output calculated from Aguilera [14] as 10.32% of digestible energy; ^8^ ME = digestible energy—urine energy—methane energy.

**Table 6:** Effects of experimental diets ^1^ on blood biochemical parameters of goats in late lactation (150 to 180 days).

Parameter (Units) ^2^	Late Lactation ^1,3^			SEM	*p* ^4^
	Control	DOP40	DOP80		
Total proteins, g/L	80.1	87.8	85.4	3.8	ns
Albumin, g/L	30.9	35.5	34.2	1.5	ns
Triglycerides, mmol/L	0.30	0.31	0.32	0.01	ns
Cholesterol, mmol/L	1.96	2.16	1.97	0.08	ns
Glucose, mmol/L	3.96	4.32	3.95	0.09	ns
Urea nitrogen, mmol/L	4.17	4.97	4.28	0.29	ns
Creatinine, µmol/L	54.1	56.9	56.6	2.2	ns
Total bilirubin, µmol/L	4.19a	2.38b	2.51b	0.41	*
Alanine aminotransferase, IU/L	10.4	12.0	15.0	1.4	ns
Aspartate aminotransferase, IU/L	90.2	69.8	73.6	5.2	ns
Alkaline phosphatase, IU/L	204.9	240.3	84.0	41.2	ns
Creatine kinase, IU/L	238.2a	153.7b	144.1b	20.2	*
Amylase, IU/L	51.9	36.0	42.9	5.3	ns
Phosphorus, mmol/L	2.55	2.49	1.98	0.12	ns
Total calcium, mmol/L	2.75	2.25	2.18	0.09	ns
Magnesium, mmol/L	1.23	1.10	1.13	0.12	ns
Sodium, mmol/L	149.1	148.3	150.1	2.2	ns
Potassium, mmol/L	4.76	5.12	5.09	0.19	ns
Chloride, mmol/L	108.6	114.1	112.9	3.3	ns
Iron, µmol/L	28.6	23.6	27.5	1.4	ns

^1^ Control, diet based on commercial concentrates plus alfalfa hay; DOP40, diet based on concentrates with 40% of cereals replaced by dehydrated orange pulp (DOP) plus alfalfa hay; DOP80, diet based on concentrates with 80% of cereals replaced by DOP plus alfalfa hay; ^2^ for all blood parameter analyses, *n* = 10 in each group. ^3^ No effect of prolificacy or diet × prolificacy interaction was observed for any variable examined (*p* > 0.05). ^4^ Statistical probability for comparisons: ns, not significant (*p* > 0.05); *, *p* ≤ 0.05. Means with different letters (a, b, c) within each row differ significantly.

**Table 7:** Profitability of the different diets throughout lactation.

Items	Diets		
	Control	DOP40	DOP80
Average milk sales price (EUR/litre)	0.7064	0.7064	0.7064
Total average income from milk sales (EUR/goat) (TAI)	178.44	178.51	179.85
Average feed costs (EUR/goat)	81.66	80.46	79.80
All other average costs (EUR/goat)	58.78	58.78	58.78
Total average milk production costs (EUR/goat) (TAC)	140.44	139.24	138.58
Average profit (EUR/goat) (TAI–TAC)	38.00	39.27	41.27
Average profit (EUR/goat/day)	0.21	0.22	0.23
Average profit (EUR/goat/litre of milk)	0.15	0.16	0.16

## Data Availability

The data presented in this study are available on request from the corresponding autor.
